# Associations between socioeconomic status and mental health trajectories during early adolescence: Findings from the Adolescent Brain Cognitive Development study

**DOI:** 10.1002/jcv2.70001

**Published:** 2025-02-25

**Authors:** Divyangana Rakesh, John C. Flournoy, Katie A. McLaughlin

**Affiliations:** ^1^ Neuroimaging Department Institute of Psychology Psychiatry & Neuroscience King's College London London UK; ^2^ Department of Psychology Harvard University Cambridge Massachusetts USA; ^3^ Ballmer Institute University of Oregon Portland Oregon USA

**Keywords:** attention, education, externalizing, financial adversity, income, internalizing, longitudinal, mental health trajectories, neighborhood, socioeconomic status

## Abstract

**Background:**

Low socioeconomic status (SES) during childhood is associated with higher levels of youth psychopathology. However, limited longitudinal work has examined the role of both household and neighborhood SES in shaping mental health trajectories over time using population‐based data. The goal of the present study was to characterize associations between SES and changes in mental health problems during early adolescence.

**Methods:**

We investigated independent and joint associations of household income‐to‐needs ratio, parent educational attainment, material hardship, and neighborhood disadvantage with internalizing, externalizing, and attention symptom trajectories using longitudinal data from the Adolescent Brain Cognitive Development (ABCD) Study. Given sex‐based differences in mental health trajectories in the ABCD study, analyses were conducted separately in males and females. SES was assessed at baseline and youth‐reported mental health was assessed across six time‐points from age 10–13 years (*M* = 10.4, SD = 0.63 years; first assessed at the 6‐month follow‐up; *N* = 9488).

**Results:**

Main effects indicated that, in general, high SES was associated with lower mental health symptoms. However, longitudinally, lower SES was associated with lower increases in mental health problems over time relative to higher SES. In females, while internalizing symptoms increased at all levels of income‐to‐needs, the association was most positive at higher levels of income‐to‐needs (*B* = 0.036, SE = 0.008, *p* < 0.001). In males, income‐to‐needs positively predicted externalizing (*B* = 0.022, SE = 0.007, *p* = 0.002) and attention (*B* = 0.023, SE = 0.007, *p* = 0.001) symptom trajectories, with lower income‐to‐needs linked to lower increases in externalizing and attention symptoms relative to higher income‐to‐needs. Two‐way interactions between SES indicators predicting changes in symptoms were non‐significant.

**Conclusion:**

Our finding that youth from lower‐SES backgrounds exhibited lower increases in mental health problems during early adolescence contrasts with findings from prior cross‐sectional studies. However, mental health problems are on the rise and the landscape of risk for psychopathology is changing. More research is needed to understand how childhood SES contributes to risk and resilience for psychopathology during the transition to adolescence.

## Graphical abstract

High SES was associated with lower mental health symptoms cross‐sectionally. However, longitudinally, higher SES was associated with greater increases in mental health problems over time. A higher income‐to‐needs ratio predicted greater increases in internalizing, externalizing, and attention problems.

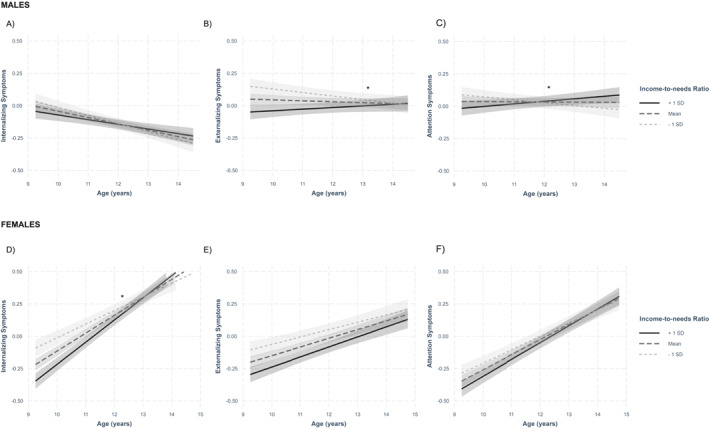

**Key points**

**What's known?**

- Low socioeconomic status (SES) is associated with higher mental health problems during adolescence. However, our knowledge of how different indicators of SES influence longitudinal trajectories of mental health is limited.

**What's new?**

- In a large sample of U.S. adolescents, lower SES across numerous indicators was associated with higher mental health problems cross‐sectionally but lower *increases* in mental health problems from 10 to 13 years.

**What's relevant?**

- Mental health problems are on the rise and the landscape of risk for psychopathology is changing. The COVID‐19 pandemic may have altered how SES influences the emergence of mental health problems during early adolescence.

## INTRODUCTION

Low socioeconomic status (SES) during childhood is associated with higher levels of youth internalizing, externalizing, and attention symptoms (McLaughlin et al., 2011; McLaughlin, Costello, et al., 2012; Peverill et al., 2021; Reiss, 2013; Russell et al., 2016). Systematic reviews and meta‐analyses demonstrate that children from socioeconomically disadvantaged backgrounds are two to three times more likely to develop mental health problems than their more advantaged peers (Peverill et al., 2021; Reiss, 2013). This relationship exists across different measures of SES, such as parental income, education, and neighborhood‐level disadvantage. Further, SES disparities in youth psychopathology persist even after controlling for other types of adverse childhood experiences (McLaughlin et al., 2011), suggesting that SES plays a unique role in shaping mental health in young people. However, longitudinal work that considers the role of different measures of SES in shaping mental health *trajectories* has been limited, particularly in population‐based samples. Given that mental health problems contribute significantly to global burden of disease in youth (Whiteford et al., 2013) and frequently have their onset during adolescence (Solmi et al., 2022), understanding how SES influences the onset and development of psychopathology during this period can inform strategies aimed at reducing mental health disparities in young people.

SES is a complex construct that can be measured in numerous ways that reflect access to both economic and non‐economic resources. Common ways to measure SES in young people include parent income or income‐to‐needs ratio, educational attainment, and neighborhood disadvantage—typically measured through neighborhood‐level income, education, and employment levels (Farah, 2017). These different metrics of SES are only moderately correlated (Oakes & Rossi, 2003) and may represent distinct aspects of the environment that influence psychopathology through different pathways (Braveman et al., 2005; Chen & Paterson, 2006; Peverill et al., 2021). The income‐to‐needs ratio reflects access to financial resources. Low income‐to‐needs can place constraints on parent's time and resources and can thus be associated with access to nutrition, housing, and other basic necessities; availability and quality of childcare; and the ability to provide stimulating learning opportunities both in and out of the home. In addition, direct measures of financial strain may provide additional information about material hardship as differences in location, expenses, lifestyle, and other responsibilities make income, even when adjusted for family size, only a cursory measure of income relative to need (Kahn & Pearlin, 2006). Parental educational attainment may be associated with cognitive stimulation, linguistic input, and the duration and quality of parent‐child interactions (Duncan & Magnuson, 2012), which are in turn associated with children's outcomes (Rakesh, Lee, et al., 2024). Neighborhood SES, which is often an aggregate of neighborhood level income, employment, and education, captures distinct characteristics, such as access to green space and exposure to crime, violence, and pollution, may also influence development and mental health (Evans, 2004). Therefore, in addition to household SES, examining the role of neighborhood SES in adolescent mental health trajectories is of crucial importance.

While income, educational attainment, and neighborhood disadvantage have all been linked to higher levels of psychopathology during childhood and adolescence, these different socioeconomic indicators may influence mental health in unique ways. Indeed, different measures of SES have been shown to have distinct associations with type of psychopathology as well as with the onset, persistence, and severity of different mental health problems (McLaughlin, Costello, et al., 2012; McLaughlin, Green, et al., 2012), but findings at the level of the specific SES indicator are mixed. For example, previous work has found higher parent education, but not income or relative deprivation, to be associated with lower risk for anxiety disorders (McLaughlin, Costello, et al., 2012). It has also been shown that material hardship, operationalized as food insecurity, but not income or community‐level inequality, is associated with mental health across domains (McLaughlin, Green, et al., 2012). In contrast, findings based on a large sample of adolescents suggest that material hardship and poverty status, but not education, are associated with depression and anxiety (Edmunds & Alcaraz, 2021). However, both income and education have also been shown to play a role in adolescent mental health in some studies (Kinge et al., 2021; Thomson et al., 2017).

Further, differential associations of various SES measures with youth psychopathology were also reported in a recent meta‐analysis of population‐based studies from the United States (Peverill et al., 2021). The study revealed that specific SES indicators, such as receiving public assistance, demonstrated stronger correlations with child psychopathology compared to others. Furthermore, this meta‐analysis found SES to be more strongly associated with childhood externalizing problems than with internalizing psychopathology (Peverill et al., 2021). Longitudinally, one study revealed that neighborhood SES did not correlate with trajectories of depressive symptoms from adolescence to adulthood (Barr, 2018). However, this study did not consider the role of household income and focused solely on depression as an outcome, leaving unanswered questions regarding the influence of various SES indicators on different mental health outcomes over time. Therefore, further research is needed to clarify associations between SES and mental health during adolescence. Indeed, no research to our knowledge has specifically examined associations between these different SES metrics (i.e., parent income, education, neighborhood disadvantage, and material deprivation) and the longitudinal development of mental health symptoms during adolescence, particularly using socioeconomically and ethnically diverse population‐based data, limiting understanding of whether associations between SES and mental health persist, diminish, or magnify across development. This is particularly important to examine during adolescence, a developmental period when risk for psychopathology increases dramatically (Solmi et al., 2022).

Some have argued that different SES indicators may interact in predicting child outcomes under a protective, additive, or mismatch framework (Ackerman & Brown, 2006; Gordon et al., 2003; Kupersmidt et al., 1995; Morrissey & Vinopal, 2018). Specifically, some models predict that more advantaged neighborhoods should lead to improved outcomes for children from low household SES but not high household SES backgrounds (Ackerman & Brown, 2006; Gordon et al., 2003; Kupersmidt et al., 1995; Morrissey & Vinopal, 2018). Other models predict that a mismatch between children's home and neighborhood environments should predict poorer outcomes (Ackerman & Brown, 2006; Gordon et al., 2003; Kupersmidt et al., 1995; Morrissey & Vinopal, 2018). A final model suggests that family and neighborhood income contribute independently and additively to children's outcomes (Ackerman & Brown, 2006; Gordon et al., 2003; Kupersmidt et al., 1995; Morrissey & Vinopal, 2018), similar to a cumulative risk framework (Evans et al., 2013). Interactions between different SES indicators in predicting youth mental health have previously been found in cross‐sectional studies. For example, McLaughlin and colleagues (2012) found a significant interaction between parental education and subjective social status in predicting externalizing disorders; higher subjective status was associated with lower odds of externalizing disorders for most respondents, except for those with parents who had the lowest education level. Similarly, Weinberg et al. (2019) showed that high subjective SES has a protective effect on the negative association between parental SES and adolescent mental health problems. Whether such interactions exist for absolute measures of SES remains unknown, particularly using longitudinal data.

The goal of this study was to investigate associations of different SES metrics—including income‐to‐needs, parent educational attainment, material hardship, and neighborhood disadvantage—with changes in symptoms of internalizing and externalizing psychopathology and attention problems during early adolescence using longitudinal data from the Adolescent Brain Cognitive Development (ABCD) Study. A recent meta‐analysis of population‐based studies suggests that all indicators of low SES are likely to be associated more strongly with externalizing than internalizing symptoms, however they did not specially examine changes in symptoms over time (Peverill et al., 2021). As such, we expected low SES to be associated with greater increases in mental health problems over time. Given the paucity of longitudinal literature that has considered both household and neighborhood SES and change in mental health symptoms, we refrained from forming concrete hypotheses about associations between specific SES indicators and mental health domains. We additionally examined interactions between the SES indicators in predicting mental health trajectories.

## METHODS

Analysis plans were pre‐registered on the Open Science Framework (https://osf.io/w5kug) (Saragosa‐Harris et al., 2022; Weinberg et al., 2019). All deviations from the pre‐registration have been fully described.

## PARTICIPANTS

Participants were from the ongoing ABCD Study (https://abcdstudy.org/; release 5). The ABCD study is a large multi‐site longitudinal study which has recruited over 11,800 children (aged 9–10 years) in order to comprehensively characterize psychological and neurobiological development from early adolescence to young adulthood (Garavan et al., 2018). All parents submitted their written, informed consent, and all children gave their consent. The rights of participants were protected under the local institutional review boards.

The present study utilized data from the baseline and 6‐month, 1‐year, 18‐month, 2‐year, 30‐month, and 3‐year post baseline time points. Baseline data were collected from 2016 to 2018 and the 2‐year follow‐up data were collected in 2018‐2020, when the COVID‐19 pandemic started. After excluding for missing data on any of the four SES variables at baseline, the final sample for the main analysis consisted of 9721 participants who had complete baseline SES data as well as data on internalizing, externalizing, and/or attention symptoms at a minimum of one time point. The sample consisted of 9488 (4521 females) at the second time point (i.e., 6 months post baseline when self‐reported mental health was first assessed). The sample size and demographic information at each time point is provided in Table 1 and differences in baseline SES and mental health at the second time point (6‐month follow‐up when self‐reported mental health was first assessed) between those who dropped out versus those who didn't are provided in the Supporting Information S1: Table S1).

**TABLE 1:** Sample size and demographic information (mean and standard deviation) at each time point for participants that had data for all four SES variables at baseline.

Follow up time‐point	6 months	1‐year	18 months	2‐year	30 months	3‐year
N (females)	9488 (4521)	9357 (4468)	9276 (4414)	9186 (4368)	8635 (4130)	8712 (4140)
Age in years	10.4 (0.63)	10.93 (0.64)	11.4 (0.64)	12.03 (0.67)	12.41 (0.64)	12.91 (0.65)
Internalizing	1.78 (2.06)	1.72 (2.1)	1.52 (1.92)	1.8 (2.21)	1.81 (2.28)	2.02 (2.38)
Externalizing	1.92 (1.98)	1.96 (1.97)	1.75 (1.87)	2.07 (1.98)	1.88 (1.9)	2.08 (1.98)
Attention	3.14 (2.52)	3.15 (2.66)	2.87 (2.5)	3.3 (2.69)	3.14 (2.61)	3.58 (2.75)
Neighborhood advantage	62.3 (26.2)	62.25 (26.26)	62.34 (26.2)	62.27 (26.18)	63.13 (25.74)	62.86 (25.97)
Educational attainment	15.34 (2.5)	15.35 (2.5)	15.36 (2.49)	15.36 (2.49)	15.4 (2.48)	15.42 (2.47)
Income‐to‐needs ratio	3.71 (2.39)	3.72 (2.39)	3.73 (2.39)	3.71 (2.38)	3.77 (2.38)	3.76 (2.38)
Low material hardship	6.57 (1.06)	6.57 (1.06)	6.57 (1.05)	6.57 (1.06)	6.59 (1.04)	6.58 (1.04)

*Note*: Values of baseline SES have been reported for participants at each time point. This table reports recoded values of ADI (as neighborhood advantage) and material hardship (as low material hardship).

## MEASURES

### Socioeconomic status

SES was assessed at baseline (age 9–10 years). The income‐to‐needs ratio was calculated as total household income (i.e., median value of the income band) divided by the federal poverty line for a family of that size. A value of 1 reflects income exactly at the poverty threshold and values greater than and less than 1 reflect being above and below the threshold, respectively). Income‐to‐needs was not calculated for individuals missing either household income or household size. We calculated parent educational attainment as the average education for both parents (in years). Data for one parent was used when data for both parents was unavailable. Finally, we utilized the Parent‐Reported Financial Adversity Questionnaire (PRFQ; sum of seven items) to assess material hardship. The PRFQ asks seven yes or no questions about ability to afford basic needs (e.g., food, health care, and shelter). Individuals missing > 30% items on PRFQ were not included in analyses including PRFQ. The PRFQ was reverse coded so higher values indicate lower material hardship. We used a composite measure of neighborhood disadvantage based on the participants' primary address: the area deprivation index (ADI). ADI was calculated at the census‐tract level based on 17 different factors including employment, education, income, and housing quality. ADI provides rankings of neighborhoods by socioeconomic disadvantage as a national percentile (Kind et al., 2014; Singh, 2003). ADI values were reverse coded so higher values indicate lower disadvantage. This variable is therefore referred to as neighborhood advantage in this manuscript. See the Supporting Information S1: Figure S1 and Figure S2 for correlations between SES variables and distributions, respectively.

### Mental health

We utilized youth‐reported symptoms of psychopathology on the Brief Problem Monitor (BPM). Outcome variables included scores on the three BPM subscales: internalizing, externalizing, and attention problems. Youth‐reported mental health was assessed at six time points starting at 6 months following baseline. Data from all available time points were utilized to model longitudinal change in symptoms. Although not preregistered, given the skewed nature of the data, we transformed the raw values based on the Yeo Johnson method for transformation (using the *bestNormalize* package in R). Distributions of transformed and untransformed values at each time point and plots for transformed versus non‐transformed values are provided in Supporting Information S1: Figure S3 and S4, respectively. Model residuals with both transformed values and raw values as well as model output with raw values have been included in the Supporting Information S1: Figure S5).

### Statistical analysis

To examine associations between SES and mental health trajectories, we ran linear mixed models using the *lme4* package in R with scores on the BPM subscales as time varying dependent variables (six time‐points; 6‐month, 1‐year, 18‐month, 2‐year, 30‐month, and 3‐year post baseline) using three separate models for each outcome (i.e., internalizing, externalizing, and attention symptoms). Predictors included time‐invariant measures of baseline SES (i.e., income‐to‐needs ratio, parent educational attainment, material hardship, and ADI), age (modeled as time‐varying; six time‐points), and interactions between each SES variable and age. This interaction term between SES and age allowed us to examine associations of SES with change in mental health symptoms over time (i.e., the slope). Age was standardized for analyses. In addition, to interpret associations between SES and slope, we additionally examined associations of SES with mental health cross‐sectionally (i.e., the intercept). To do this, we also report the main effects of SES from the previously described models (i.e., models with SES*age interactions predicting time varying mental health). Since age was standardized, the main effect SES extracted from the model represents associations of SES with mental health at the mean age across time points. The *lmerTest* package was used to obtain *p* values. Importantly, although not preregistered, given sex differences in mental health trajectories in this sample (see Supporting Information S1: Figure S6), and sex‐differences in vulnerability to different types of psychopathology (Martel, 2013), we report results separately for males and females. Instead of a full model with numerous three‐way interactions between each SES indicator, age, and sex, we opted for sex‐stratified analyses. The complexity of the full interaction model would make it challenging to interpret specific SES effects due to the overlapping contributions of each indicator, as well as the potential for multicollinearity. By stratifying the analyses, we simplify the model, reduce multicollinearity, and provide clearer insights into the unique impacts of SES indicators on mental health trajectories within each sex. For transparency, we report findings across the whole sample in the Supporting Information S1: Table S2).

Race/ethnicity has previously been shown to be associated with mental health trajectories (Akee et al., 2019; McLaughlin et al., 2007; Nazroo et al., 2020; Platt et al., 2021). As such, race/ethnicity was included as a covariate in our models. However, since effects of race/ethnicity and disadvantage can be difficult to disentangle (Meghani & Chittams, 2015), model output without race/ethnicity as a covariate is included in the Supporting Information S1: Table S3 and S4). Lower‐order main effects were automatically included in the model. Subject and family were modeled as random effects. Site was not modeled as a random effect as it accounted for minimal variance and contributed to model convergence issues. See the Supplement for model equations. Because we estimated associations between SES and three separate mental health outcomes, findings were considered significant at *p* < 0.0167. For meta‐analytic purposes we tested independent models (where the interaction between age and only one SES variable was included at a time; reported in the Supporting Information S1: Table S5). Further, in sensitivity analyses for significant effects we included the relevant SES variable (interacting with age) as the sole predictor in the model to ensure that the inclusion of covariates did not impact findings. See the Supplement for model equations.

To examine interactions between individual and neighborhood SES as described in the introduction, we added the following interactions (with age) to the models described above: i) income to needs ratio and ADI, ii) educational attainment and ADI, and iii) material hardship and ADI. In addition, to evaluate whether one form of SES buffers or magnifies the associations of other aspects of SES with mental health, we also examined interactions between i) income‐to‐needs and educational attainment and ii) educational attainment and material hardship. Lower‐order main and interaction effects were also included in the model. See the Supplement for model equations. We accounted for multiple comparisons using False Discovery Rate correction at *p*FDR < 0.0167 (to account for 15 models run across three outcomes).

*Non‐preregistered supplemental analyses*. To aid with interpreting our results, we conducted additional analyses that examine associations of SES with COVID‐19 related changes that may influence mental health. Specifically, we examined associations of SES with self‐reported COVID‐19 life impact and changes in familial and social relationships. In addition, we also examined parent involvement in schoolwork as a measure of support. These additional data were gathered through a dedicated COVID‐19 sub‐study conducted during the pandemic period. Given that these analyses were not preregistered and were conducted solely to provide context for our results, we have included them in the Supplementary Materials and briefly addressed them in the Discussion to bolster our interpretation.

## RESULTS

### Demographic information

Demographic information (at each time point) is available in Table 1.

### SES and mental health trajectories over time

**FIGURE 1:**
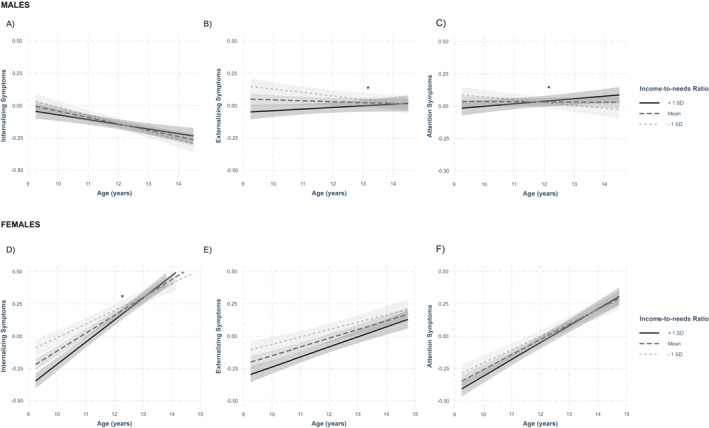
Associations between income‐to‐needs and trajectories of internalizing (A), externalizing (B), and attention (C) problems in males during adolescence. Associations between income‐to‐needs and trajectories of internalizing (C), externalizing (D), and attention (E) problems in females during adolescence. The *y*‐axis represents transformed values. To enhance visibility of slopes, the *y*‐axis scale has been reduced.

Our main analysis modeled interactions between SES and age to characterize associations of SES with change in mental health trajectories over time (i.e., the slope). When income‐to‐needs, neighborhood advantage, educational attainment, and material hardship were included in the same model, only income to needs was associated with mental health trajectories in both males and females (Figure 1; Table 2). Specifically, in females, while internalizing symptoms increased at all levels of income‐to‐needs, the association was most positive at higher levels of income‐to‐needs. That is, a lower income‐to‐needs ratio was associated with *lower* symptom increases over time relative to higher income‐to‐needs (*B* = 0.036, SE = 0.008, *p* < 0.001).

**TABLE 2:** Model output.

Mental health variable	SES variable	B	SE	T	*p*	
Males						
Internalizing	Income‐to‐needs	0.014	0.007	1.963	0.050	
	Education	0.011	0.007	1.538	0.124	
	Low material hardship	−0.001	0.006	−0.170	0.865	
	Neighborhood advantage	0.004	0.007	0.576	0.565	
Externalizing	Income‐to‐needs	0.022	0.007	3.113	0.002	*
	Education	−0.009	0.007	−1.328	0.184	
	Low material hardship	−0.003	0.006	−0.465	0.642	
	Neighborhood advantage	0.001	0.007	0.217	0.828	
Attention	Income‐to‐needs	0.023	0.007	3.409	0.001	*
	Education	0.005	0.007	0.771	0.440	
	Low material hardship	−0.002	0.005	−0.335	0.737	
	Neighborhood advantage	0.003	0.006	0.412	0.680	
Females						
Internalizing	Income‐to‐needs	0.036	0.008	4.658	<0.001	*
	Education	0.003	0.008	0.448	0.654	
	Low material hardship	0.002	0.006	0.345	0.730	
	Neighborhood advantage	0.015	0.007	2.159	0.031	
Externalizing	Income‐to‐needs	0.011	0.007	1.521	0.128	
	Education	−0.002	0.007	−0.255	0.799	
	Low material hardship	0.007	0.006	1.154	0.248	
	Neighborhood advantage	0.002	0.007	0.257	0.797	
Attention	Income‐to‐needs	0.015	0.007	2.040	0.041	
	Education	0.002	0.007	0.298	0.766	
	Low material hardship	0.000	0.006	0.022	0.983	
	Neighborhood advantage	0.008	0.007	1.191	0.234	

*Note*: Model output for associations between SES indicators and internalizing, externalizing, and attention symptom trajectories. Results are from models where all four SES indicators were included simultaneously. The table reports uncorrected *p* values. *p < 0.0167.

In males, income‐to‐needs ratio was positively associated with trajectories of externalizing (*B* = 0.022, SE = 0.007, *p* = 0.002) and attention (*B* = 0.023, SE = 0.007, *p* = 0.001) symptoms. A test of the simple slopes showed that the association between lower income‐to‐needs ratio and decreases in externalizing symptoms (i.e., negative slope) was significant. For attention symptoms, a test of the simple slopes showed that the association between higher income‐to‐needs and greater increases in symptoms (i.e., positive slope) and a lower income‐to‐needs and greater decreases in symptoms (i.e., negative slope) were both significant. See the Supplement for results of the test of the simple slopes.

All indicators except material hardship (in males) were associated with changes in mental health symptoms in independent models (where only one SES variable was included at a time; see Table S3). Further, sensitivity analyses revealed that results remained unchanged when INR was included in the model as the sole predictor (model output available in the Supporting Information S1: Table S6). Analyses with raw (i.e., untransformed) values also revealed higher INR to be positively associated with changes in internalizing symptoms in females and externalizing and attention symptoms in males (Table S7).

### Main effects of SES on mental health symptoms

To clarify the association with SES and overall mental health in the sample cross‐sectionally, we additionally report the main effects of SES (i.e., the intercept). Since age was standardized for analyses, the coefficients for the main effects effectively represent the cross‐sectional association of SES with mental health at the mean age across time points. In males, main effects suggest that income‐to‐needs ratio was negatively associated with externalizing symptoms. Parent educational attainment was negatively associated with internalizing and attention symptoms. Neighborhood advantage and lower material hardship were negatively associated with mental health problems across all three domains. In females, main effects suggested that income‐to‐needs ratio was negatively associated with internalizing and externalizing symptoms. Parent educational attainment was negatively associated with attention symptoms. Neighborhood advantage was negatively associated with externalizing and attention symptoms and lower material hardship was negatively associated with mental health problems across all three domains. See Table 3 for model output.

**TABLE 3:** Model output for main effects for males and females.

Mental health variable	SES variable	B	SE	T	*p*
Males					
Internalizing	Income‐to‐needs	−0.008	0.014	−0.538	0.590
	Education	−0.036	0.014	−2.510	0.012
	Low material hardship	−0.074	0.012	−6.374	<0.001
	Neighborhood advantage	−0.042	0.013	−3.162	0.002
Externalizing	Income‐to‐needs	−0.052	0.015	−3.390	0.001
	Education	−0.018	0.015	−1.206	0.228
	Low material hardship	−0.095	0.012	−7.717	<0.001
	Neighborhood advantage	−0.045	0.014	−3.192	0.001
Attention	Income‐to‐needs	−0.002	0.016	−0.144	0.886
	Education	−0.052	0.015	−3.373	0.001
	Low material hardship	−0.095	0.013	−7.598	<0.001
	Neighborhood advantage	−0.032	0.015	−2.180	0.029
Females					
Internalizing	Income‐to‐needs	−0.049	0.016	−3.066	0.002
	Education	−0.010	0.016	−0.656	0.512
	Low material hardship	−0.072	0.013	−5.596	<0.001
	Neighborhood advantage	−0.023	0.014	−1.576	0.115
Externalizing	Income‐to‐needs	−0.071	0.016	−4.370	<0.001
	Education	0.001	0.016	0.051	0.960
	Low material hardship	−0.047	0.013	−3.500	<0.001
	Neighborhood advantage	−0.048	0.015	−3.252	0.001
Attention	Income‐to‐needs	−0.029	0.017	−1.694	0.090
	Education	−0.050	0.017	−3.034	0.002
	Low material hardship	−0.069	0.014	−4.966	<0.001
	Neighborhood advantage	−0.039	0.015	−2.542	0.011

*Note:* Model output for the main effects of SES indicators in models predicting internalizing, externalizing, and attention symptom trajectories. Results are from models where all four SES indicators were included simultaneously. The table reports uncorrected *p* values.

### Joint associations between SES and mental health trajectories

We found no significant joint associations of SES indicators with mental health trajectories. Model output is available in the Supporting Information S1: Table S8).

## DISCUSSION

The aim of this study was to characterize independent and joint associations between SES and trajectories of internalizing, externalizing, and attention symptoms from 10 to 13 years in a large national sample of adolescents. High SES was associated with lower mental health problems cross‐sectionally. However, in contrast to our hypotheses, lower income‐to‐needs was associated with lower increases in mental health symptoms. Over the past few years, a period which included the global COVID‐19 pandemic, we observe the opposite association between SES and youth mental health than has typically been observed in longitudinal (Lansford et al., 2019) and population‐based cross‐sectional studies (Peverill et al., 2021). We speculate that either the pandemic or other recent changes in the landscape of child mental health may have, at least temporarily, altered the role that income plays in the emergence of psychopathology during adolescence.

To help interpret cross‐sectional differences, we examined associations between SES at baseline and mental health symptoms at the 6‐month follow up (reported in the Supporting Information S1: Table S9) as well as at the mean age across time points (i.e., the intercept; reported in the Results). We found that, in general, SES (across indicators) was negatively associated with mental health symptoms cross‐sectionally at the 6‐month follow‐up and the mean age across domains (barring a few exceptions). Our findings suggest that in general, higher financial and non‐financial resources at the household and neighborhood level are associated with lower mental health problems in early adolescence in both males and females. These results align with prior meta‐analytic work on population data and systematic reviews that have similarly shown that high SES is associated with lower risk for psychopathology (Peverill et al., 2021; Reiss, 2013). There are several factors that may contribute to these associations. For example, greater financial stability as a function of higher household SES may contribute to lower stress and anxiety (Santiago et al., 2011). Further, higher SES is often linked to better educational opportunities for children such as school quality, enrichment programs, and extra‐curricular activities, which can promote cognitive and emotional development (Bradley & Corwyn, 2002; Rakesh, McLaughlin, et al., 2024; Raniti et al., 2022). Higher SES is also associated with lower exposure to adverse childhood experiences such as violence, neglect, and family instability, which are well‐documented risk factors for developing mental health problems (Hughes et al., 2017). Finally, high SES neighborhoods usually offer safer environments, better recreational facilities, more opportunities for social engagement, and greater social support systems, all of which may buffer stress and contribute to positive mental health outcomes (Cohen & Wills, 1985; Leventhal & Brooks‐Gunn, 2000).

Given the vast literature showing links between low SES and greater mental health problems (Peverill et al., 2021; Reiss, 2013), we expected low SES to be associated with greater increases in psychopathology over time. However, although higher income‐to‐needs was associated with lower symptoms cross‐sectionally, the longitudinal trajectories (i.e., slopes) tell a different story. Specifically, a lower income‐to‐needs was associated with lower increases in internalizing symptoms in females and externalizing and attention symptoms in males over time when change associated with the other SES indicators and race/ethnicity were accounted for. Similar findings of high SES being associated with greater increases in mental health problems have previously been demonstrated, but not interpreted, in the ABCD study using prior releases that had partial data available (Barch et al., 2021). Importantly, all four SES indicators were associated with mental health problems cross‐sectionally when all four variables were included in the same model. In addition, in pre‐registered supplemental analyses, in which we ran separate models for each SES measure, we found that higher SES across all indicators (except material hardship in males) was associated with change in mental health symptoms. These findings are consistent with prior cross‐sectional work showing low SES, across different measures of SES, to be associated with worse mental health problems (Peverill et al., 2021; Reiss, 2013). However, considering the paucity of longitudinal literature, the finding that neighborhood advantage, material hardship, and parent educational attainment did not predict trajectories of mental health symptoms over time when change associated with other SES variables were accounted for, is harder to interpret. To our knowledge, the longitudinal studies that examined associations between SES and changes in symptoms (e.g., Barr, 2018; Lansford et al., 2019; Reiss et al., 2019), did not consider the role of the same socioeconomic indicators, making it challenging to reconcile findings. However, consistent with our results, Lansford and colleagues (2019) also showed that income, but not education, was associated with changes in symptoms over time. Our findings may also be a function of the specific life period examined. It is possible that these indicators of SES influence mental health differentially during different life periods (e.g., early childhood vs. adolescence). For example, the effects of neighborhood advantage may become more evident as adolescents grow older and spend more time outside their homes (Boardman & Saint Onge, 2005; Reiss et al., 2019). Further work with data spanning across adolescence and emerging adulthood is needed.

Importantly, we observed sex differences in the association of income‐to‐needs with changes in mental health symptoms over time. Specifically, while income‐to‐needs was associated with trajectories of internalizing symptoms in females, it was associated with externalizing and attention symptoms in males. These findings are broadly consistent with sex differences in psychopathology during this age period. Research consistently shows that males are generally more likely to exhibit externalizing and attention symptoms, such as those seen in attention‐deficit/hyperactivity disorder, oppositional‐defiant disorder, and conduct disorder, with a prevalence about three times higher than in females (Martel, 2013; Seedat et al., 2009). In contrast, females are more prone to developing internalizing symptoms, such as those associated with mood and anxiety disorders, which become more common during adolescence, with females being affected at roughly twice the rate of males (Martel, 2013; Seedat et al., 2009). Despite these well‐established sex differences, the underlying reasons for these differences remain poorly understood and are likely multifaceted. Possible explanations include males and females facing distinct biological risks, like prenatal hormone exposure, or responding differently to environmental and psychological stressors (Hyde et al., 2008; Martel, 2013). For example, differences in emotion regulation and greater rumination partially explain higher rates of depression and anxiety in females (Nolen‐Hoeksema, 2012). Additionally, females often experience a greater incidence of certain stressors, particularly in interpersonal contexts, and exhibit a heightened sensitivity to these stressors, resulting in more pronounced depressive symptoms (Hankin et al., 1998). The sex‐specific associations observed in the present study highlight the differential impact of SES on mental health outcomes, suggesting that income‐related stressors may influence males and females through distinct developmental pathways. However, more research is needed to fully understand the mechanisms driving these sex differences and to explore how socioeconomic factors may interact with biological and environmental influences over time. Importantly, despite these differences, the association of lower SES with reduced increase in symptoms over time was consistent across sexes: lower income‐to‐needs was associated with smaller increases in mental health symptoms across males and females. Therefore, the remainder of this Discussion focuses on the overall direction of these associations rather than the sex differences themselves, which are well‐established.

While speculative, one interpretation of higher income being associated with greater increases in symptoms relates to the potential impact of the COVID‐19 pandemic on adolescent psychopathology, during which some of the data from the later waves of the ABCD study was collected. The COVID‐19 pandemic introduced innumerable stressors into the lives of adolescents and families (Adams et al., 2021; Gadermann et al., 2021; Johnson et al., 2021; Lawson et al., 2020). Past work has shown that the experience of moderate levels of adversity may promote and foster subsequent resilience in the face of stressors (Seery et al., 2010, 2013). Specifically, results from a multiyear, national study found that individuals who had experienced some adversity in their lives had better mental health and well‐being outcomes compared to those with either a high or no history of adversity (Seery et al., 2010), a finding that has since been replicated (McGinnis, 2018). This may explain why low SES was linked to lower increases in symptoms—children from low‐income backgrounds, sometimes already facing chronic stress due to the numerous constraints that low income places on families (Evans, 2004), may have developed greater coping skills to deal with stress during the pandemic. However, given evidence showing that early‐life stress increases sensitivity to later stressors (Zhang et al., 2024), as well as work showing that the pandemic disproportionately impacted those from disadvantaged backgrounds (Chen et al., 2022), this explanation is unlikely. We provide other possible interpretations below.

Another possible explanation for our findings is that parents from low SES backgrounds (particularly in the ABCD study) may have responded to the pandemic differently than parents from high SES backgrounds in ways that contributed to lower pandemic‐related stressors or less impact of these stressors on mental health for children. For example, in the ABCD sample, families who experienced more family‐ and neighborhood‐level disadvantage reported more frequent supportive and open dialogs about COVID‐19 and ways to reduce its spread, as well as more frequent youth preventative actions than families from higher SES backgrounds (Marshall et al., 2021). Protective measures taken by these families may have reduced fear of infection with COVID‐19, thus contributing to lower increases in symptoms of stress‐related mental health problems. Further, in supplemental analyses, we showed that higher INR was positively associated with self‐reported life impact, and more negative changes in communication and general relationship quality with family, but not friends. That is, adolescents from higher income families reported that their life was more severely impacted by the pandemic. They also reported that there were more negative changes in familial communication and relationship quality. Further, higher INR was also associated with lower parent involvement in schoolwork during the pandemic. These results support the idea that parents from higher income backgrounds behaved differently than parents from lower income backgrounds, at least as perceived by their children, during the pandemic in this sample. We speculate that these differences may have contributed to our finding of lower income children experiencing lower increases in mental health problems during this time. However, it is important to note that these results may be specific to the ABCD sample and may not apply to the general population.

There are also other explanations for our findings. Mental health problems have been increasing drastically over the last decade (Twenge, 2020). Nationally representative data indicate that depressive symptoms, suicide‐related outcomes, and suicide deaths have increased markedly among American adolescents during that time (Twenge et al., 2018). Given this increase in mental health problems, it is likely that the landscape of risk for mental health problems is also changing. For example, adolescents are also spending substantially more time online and interacting through electronic devices and less time engaging in non‐screen activities like face‐to‐face social interaction, reading print media, and participating in sports or exercise; these changes have been linked to greater risk for depression and suicidal thoughts and behaviors (Twenge et al., 2018). In addition, there has also been an evolution in parenting styles in recent years, with “helicopter parenting” and over‐involved parenting styles on the rise, particularly in high‐income contexts (Doepke, 2019), which can influence children's well‐being (LeMoyne & Buchanan, 2011). This type of parenting has a differential impact based on SES, with more negative outcomes for high‐income relative to low‐income youth (McGinley & Davis, 2021). As such, changes in children's behavior and the digital landscape as well as changes in parenting behaviors may interact in new and unique ways with SES to confer risk for mental health problems. More research is needed to explore these questions. However, it is also possible that our findings may have been influenced by the nature of the attrition in the ABCD sample or regression to the mean effects. There are more high SES children present in later waves due to greater attrition from lower SES families (Table S1). Further, children who had higher mental health problems at the 6‐month time point were also more likely to have missed subsequent visits. Our results could have been influenced by this attrition; higher attrition in children from lower SES backgrounds and higher psychopathology could contribute to the observed association between lower SES and lower increases in mental health symptoms. Finally, our findings may also reflect cohort effects or be specific to the ABCD sample. More longitudinal work using other datasets is needed to test the generalizability of these findings.

Our analysis did not reveal significant interactions between the various SES indicators in predicting mental health trajectories. No other studies to our knowledge have examined the interactive effects of different SES indicators in predicting changes in mental health during adolescence, making our findings challenging to reconcile with existing literature. However, our results stand in contrast to previous cross‐sectional research by McLaughlin, Costello, et al. (2012), who identified an interaction between parental education and subjective social status when predicting externalizing disorders in young people. Their study demonstrated that while higher subjective social status generally reduced the risk of behavior problems, individuals with parents who had the lowest level of education did not experience this protective effect. It is possible that only subjective social status, which reflects an individual's own perception of their social standing and self‐worth and was not measured in the ABCD study, interacts with other absolute SES indicators in influencing mental health. It is also possible interaction effects are evident cross‐sectionally due to effects being present earlier in childhood but do not influence changes over time during early adolescence. Further, interactive effects between different SES indicators on mental health trajectories may only become evident in later life stages. For instance, an adolescent from a low‐income family might experience a protective effect from strong and supportive neighborhood connections, but such an effect may only become evident later in adolescence when they engage more with their neighborhoods—an important question to explore in future work.

Our study has several strengths, including the use of a large national sample and longitudinal data; however, findings must be considered in light of some limitations. One, some of the data of the ABCD study were collected during the COVID‐19 pandemic. Therefore, while this study provides important insight into the role that SES played in the change of mental health symptoms during this period, it may not apply outside of the pandemic context. Two, given that SES tends to be higher in ABCD relative to the national population, we cannot be sure of the generalizability of these results to the broader population. Three, given that this study was interested in the total effect of SES on mental health trajectories, we are unable to comment on the specific biological and environmental mechanisms that may have driven these associations. For example, lower income‐to‐needs is associated with earlier pubertal timing (Kelly et al., 2017) and brain structure and function (Rakesh et al., 2021a, 2022, 2023a; Rakesh & Whittle, 2021). These factors may act as mechanisms linking SES with mental health (Whittle et al., 2024). Further, due to the constrains placed on families, low income is associated with several proximal environmental factors, such as parenting style, childhood maltreatment, and school environment (Lefebvre et al., 2017; Roubinov & Boyce, 2017). These factors have been shown to, in turn, be associated with pubertal development and/or development of brain structure and function (Colich et al., 2020a, 2020b; MacSweeney et al., 2024; Rakesh et al., 2021b, 2023b, 2023c; Thijssen et al., 2017, 2022; Whittle et al., 2022) ‐ which may ultimately influence mental health outcomes (Kaltiala‐Heino et al., 2003; Whittle et al., 2024). Four, there are several factors, including supportive parenting, that may promote resilience and protect against the development of mental health problems in the context of low SES (Wilson‐Simmons et al., 2017). Understanding the interplay of these risk and protective environmental and biological factors in the associations observed in the present study need to be further explored. Five, maternal and paternal parenting may have differential associations with the mental health of male and female children (Mahadevan & Fan, 2021). Future work should aim to tease apart these associations. Finally, we are unable to determine whether these effects are transient or persistent, and future longitudinal work using ABCD data is needed.

## CONCLUSION

Lower SES was associated with lower increases in mental health problems during early adolescence in a large sample of youth. Mental health problems are on the rise and the landscape of risk for mental health is changing. More research is needed on how different risk factors may interact with SES in conferring risk and resilience for the emergence of adolescent psychopathology.

## AUTHOR CONTRIBUTIONS

**Divyangana Rakesh:** Conceptualization; formal analysis; methodology; writing–original draft; writing–review and editing. **John C. Flournoy:** Writing–review and editing. **Katie A. McLaughlin:** Supervision; writing–review and editing.

## CONFLICT OF INTEREST STATEMENT

The authors declare no conflicts of interest.

## ETHICAL CONSIDERATIONS

The need for ethics approval was waived by The University of California, Los Angeles, institutional review board (IRB) stating that secondary analyses using the publicly released ABCD Study data are not human subjects research and therefore do not require their own approval. The ABCD Study received their own central IRB approval. All guidelines pertaining to the Declaration of Helsinki were adhered to. Caregivers provided written informed consent and children provided assent for participation in the study.

## Supporting information

Supporting Information S1

## Data Availability

The ABCD Study data is available for qualified researchers who have an approved Data Use Certificate (DUC) from the National Institute of Mental Health (NIMH) Data Archive (NDA). Data used in the preparation of this article were obtained from the ABCD Study (https://abcdstudy.org) held in the NDA. This is a multisite, longitudinal study designed to recruit more than 10,000 children ages 9–10 years old and follow them over 10 years into early adulthood.
