# Supplementary material for: Associations between socioeconomic status and mental health trajectories during early adolescence: Findings from the Adolescent Brain Cognitive Development study
Source: JCPP Adv. 2025 Feb 25;5(4):e70001. doi: 10.1002/jcv2.70001 (PMC12698285; doi:10.1002/jcv2.70001)
Supplement: Supplementary file 1 — Supporting Information S1 [file JCV2-5-e70001-s001.docx]

**Supporting Information**

**Associations between socioeconomic status and mental health trajectories during early adolescence: Findings from the ABCD Study**

Divyangana Rakesh^1,2^, John C. Flournoy^2^, Katie A. McLaughlin^2,3^

^1^Neuroimaging Department, Institute of Psychology, Psychiatry & Neuroscience, King’s College London, London, UK
^2^Department of Psychology, Harvard University, USA
^3^Ballmer Institute, University of Oregon, Portland, OR, USA

**Table of Contents**

[Model Equations 3](#_Toc188264882)

[Figure S1: Distribution of SES variables 4](#_Toc188264883)

[Figure S2: Correlation values between SES and mental health outcomes at each time point for males and females 5](#_Toc188264884)

[Figure S3: Distribution of mental health variables at each time point before and after transformation 6](#_Toc188264885)

[Figure S4: Plots for transformed versus non-transformed values 7](#_Toc188264886)

[Figure S5: Model residuals before and after transformation 8](#_Toc188264887)

[Table S1: Information on changes in key variables over time due to attrition 9](#_Toc188264888)

[Table S2: Associations between SES and trajectories of mental health across the whole sample (non-sex-stratified results) 9](#_Toc188264889)

[Figure S6: Sex differences in mental health trajectories (Figure S6) 10](#_Toc188264890)

[11](#_Toc188264891)

[Simple slope results for main models 12](#_Toc188264892)

[Table S3: Model output excluding race-associated change as a covariate 15](#_Toc188264893)

[Table S4: Model output for all 3-way interactions excluding race-associated change as a covariate 16](#_Toc188264894)

[Table S5: Model output for independent effects (with only one SES indicator in the model) 18](#_Toc188264895)

[Table S6: Results of associations between income-to-needs and change in mental health (with no covariates) 21](#_Toc188264896)

[Table S7: Associations between INR and trajectories of mental health using untransformed (i.e., raw) data 21](#_Toc188264897)

[Results of associations between income-to-needs and COVID-19 impact 22](#_Toc188264898)

[Table S8: Model output for all 3-way interactions 23](#_Toc188264899)

[Table S9: Model output for the association between baseline SES and 6-month follow-up mental health symptoms 25](#_Toc188264900)

[References 26](#_Toc188264901)

## **Model Equations**

**Abbreviations for all model equations:**

INR = income-to-needs ratio

EDU = educational attainment

PRFQ = parent-reported financial adversity

ADI = area deprivation index)

Main models:

mental_health (time varying) ~ age + adi*age + inr*age + edu*age + prfq*age + race/ethnicity*age + adi + inr + edu + prfq + (1|family) + (1|subject)

3-way interaction models:

mental_health (time varying) ~ age + SES1*SES2*age + SES1*age + SES2*age + race/ethnicity*age + SES1 + SES2 + (1|family) + (1|subject)

## **Figure S1: Distribution of SES variables**

**
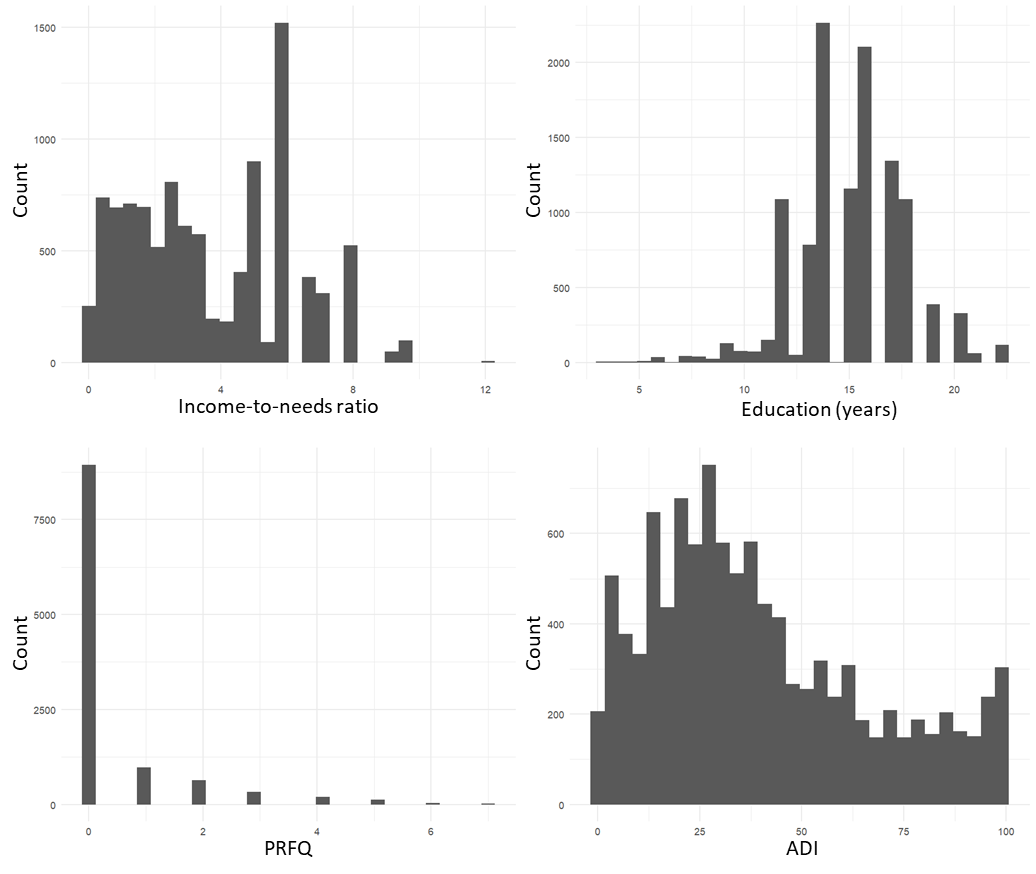
**

Figure S1. This figure plots distributions of income-to-needs ratio, education, PRFQ (parent-reported financial adversity, and ADI (area deprivation index). PRFQ and ADI values are original values (i.e., not recoded) – higher values indicate higher disadvantage.

## **Figure S2: Correlation values between SES and mental health outcomes at each time point for males and females**


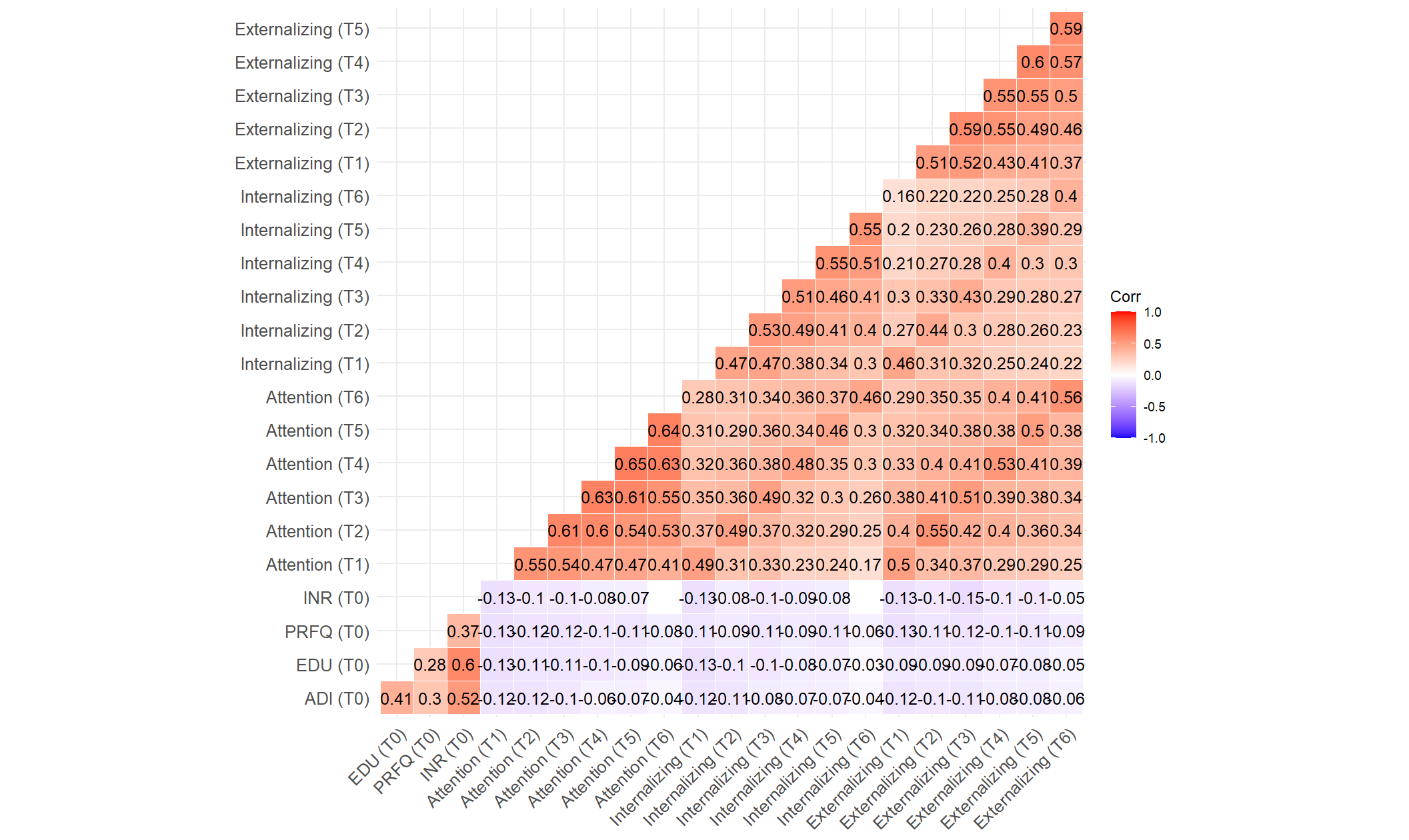

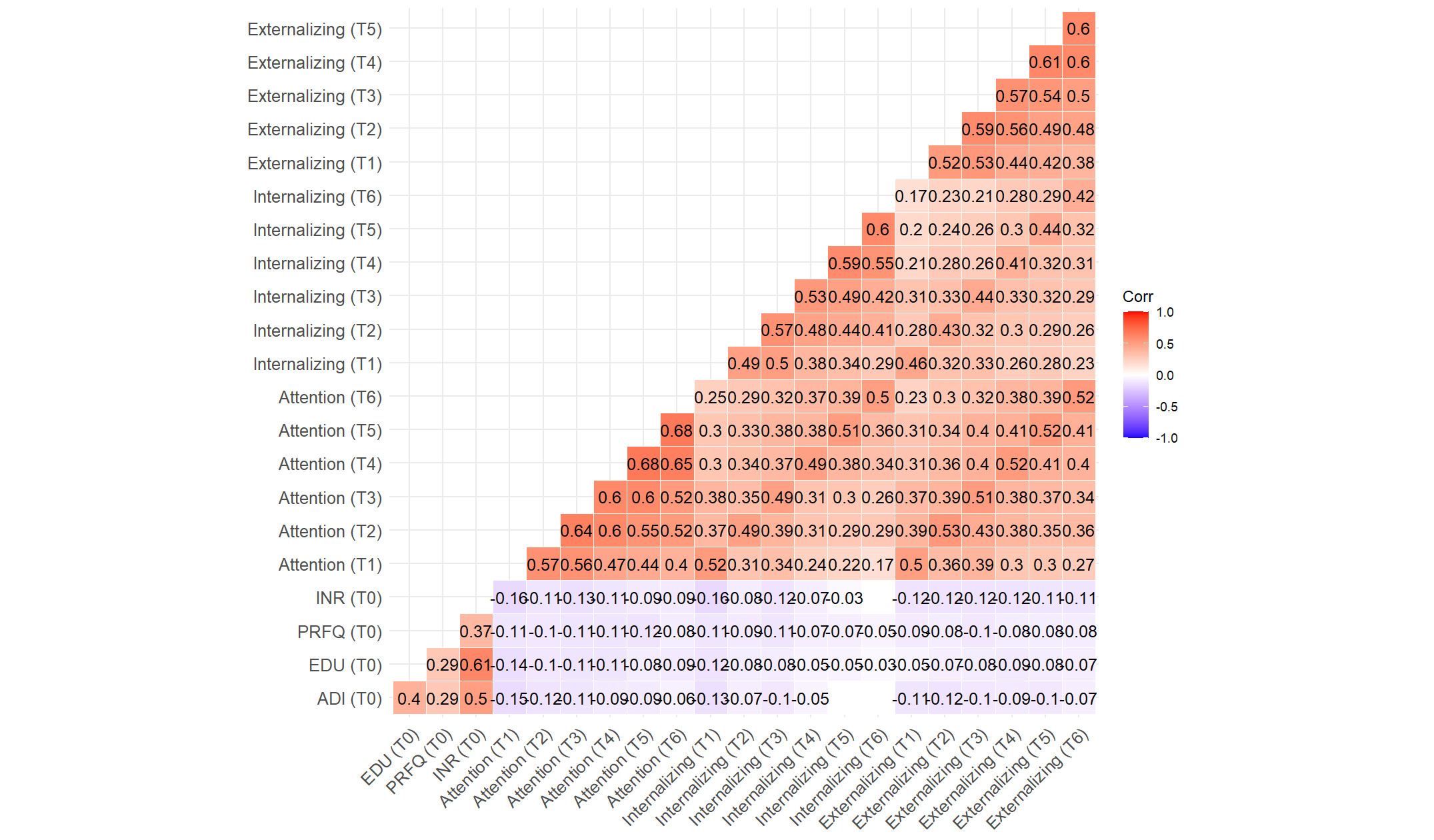


Figure S2. Correlation values were calculated with transformed values for mental health data in males (left) and females (right)

## **
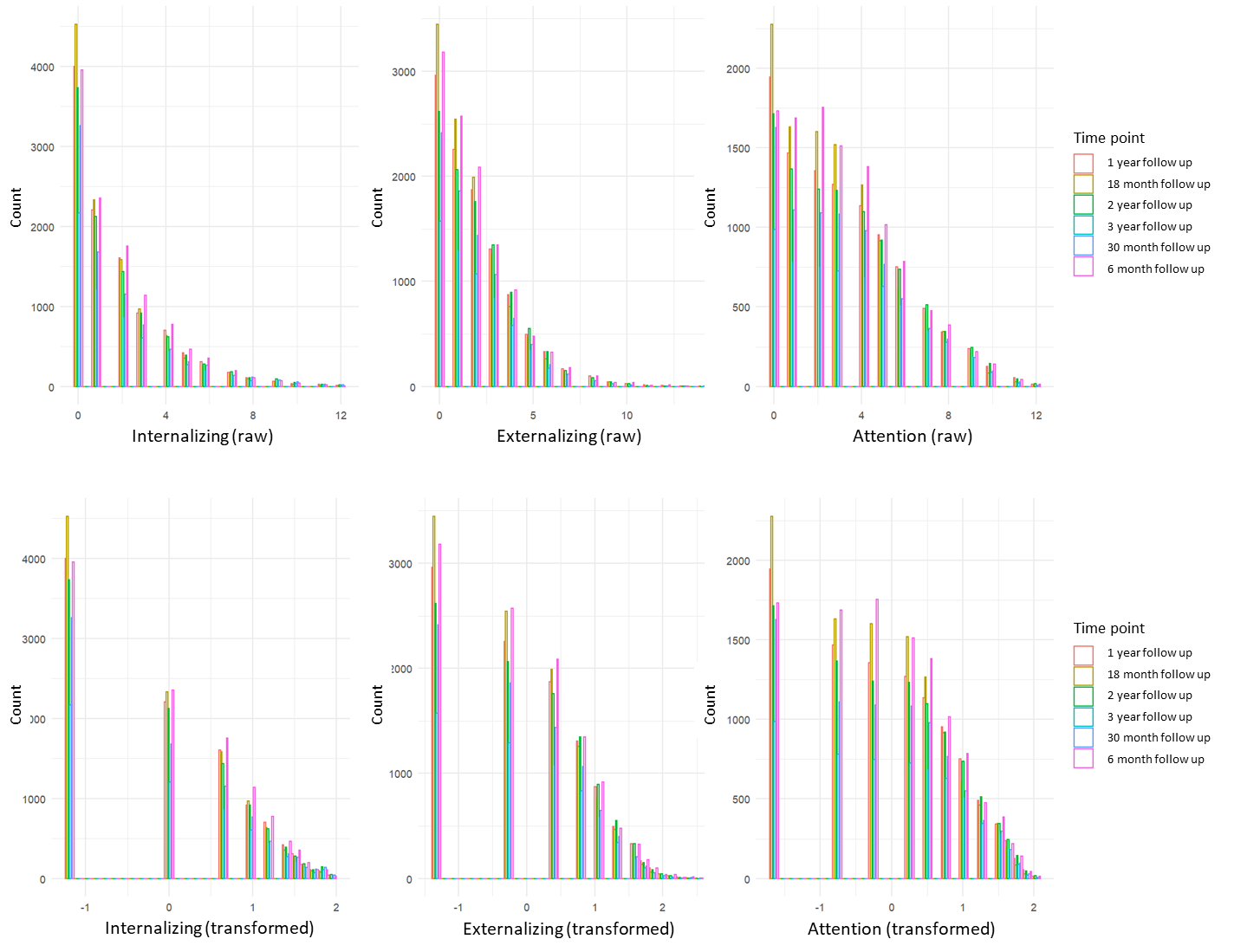
Figure S3: Distribution of mental health variables at each time point before and after transformation**

Figure S3. Top row: untransformed values**;** Bottom row: transformed values

## **Figure S4: Plots for transformed versus non-transformed values**

**
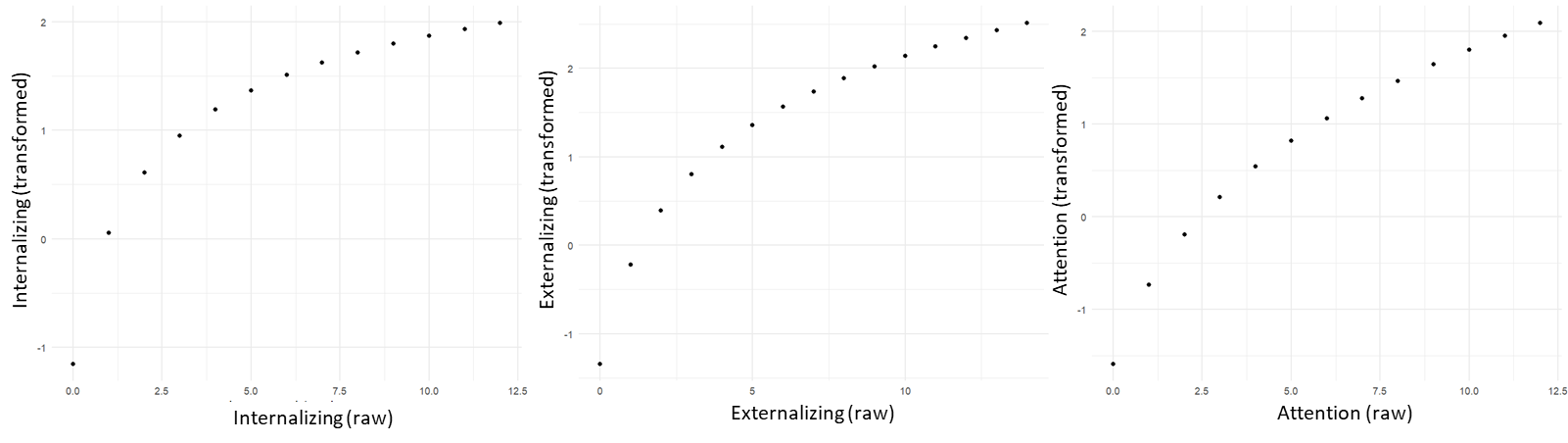
**

Figure S3

## **Figure S5: Model residuals before and after transformation**
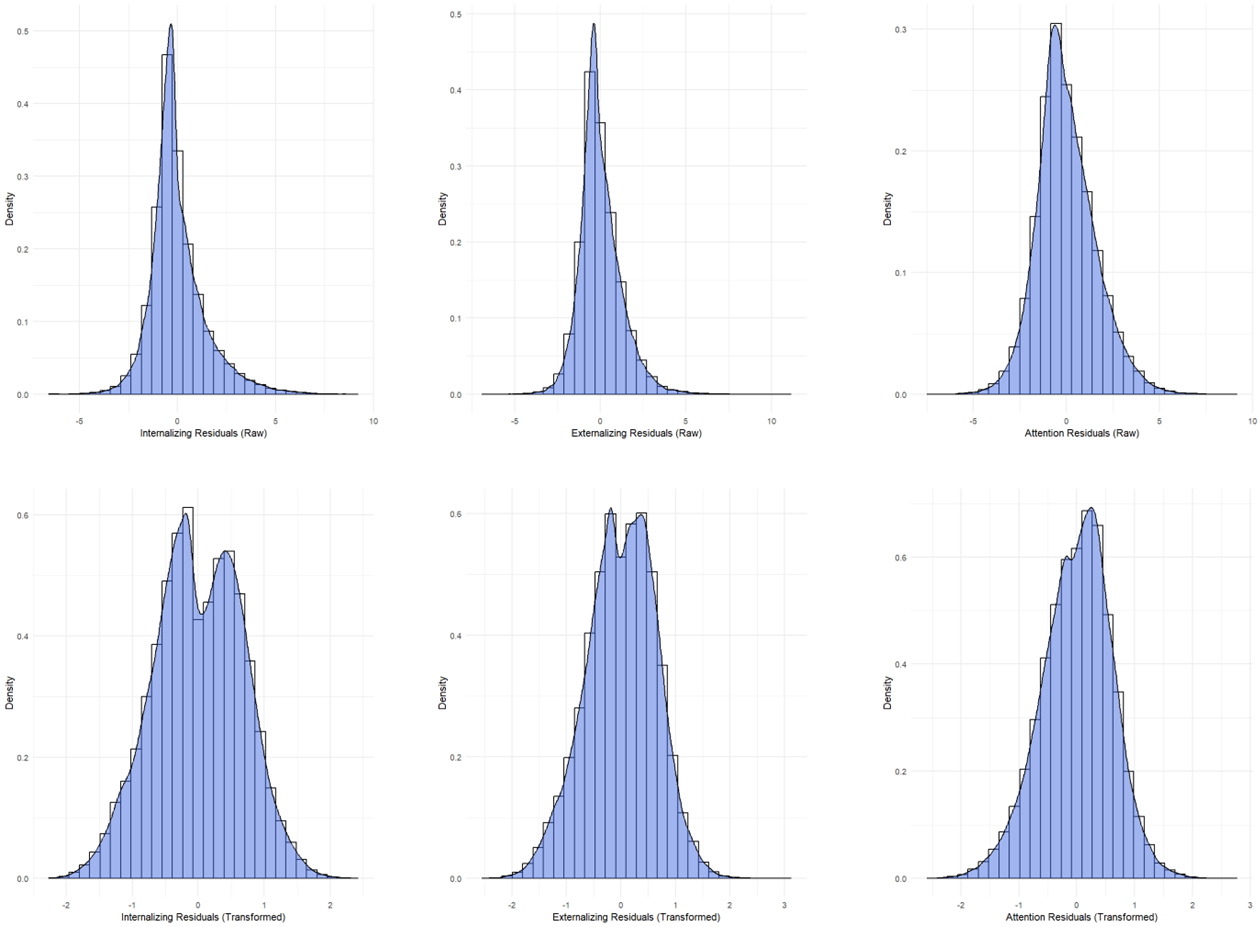


Figure S5. Top panel represents residuals before transformation and bottom panel represents residuals after transformation from models that include all four SES variables as predictors (i.e., our main models).

## **Table S1: Information on changes in key variables over time due to attrition**

|  | *1 year* | *18 months* | *2 year* | *30 months* | *3 year* |
| --- | --- | --- | --- | --- | --- |
| *Age (months)* | 0.249 (124.78, 125.26) | 0.047 (124.76, 125.53) | 0.076 (124.76, 125.38) | 0.063 (124.74, 125.21) | 0.080 (124.75, 125.2) |
| *Internalizing* | 0.302 (1.78, 1.91) | 0.226 (1.78, 1.92) | 0.3 (1.78, 1.88) | 0.003 (1.76, 1.98) | 0.003 (1.76, 1.99) |
| *Externalizing* | 0.077 (1.92, 2.14) | 0.226 (1.92, 2.05) | 0.627 (1.92, 1.97) | < 0.001 (1.89, 2.22) | < 0.001 (1.89, 2.21) |
| *Attention* | 0.085 (3.13, 3.4) | 0.512 (3.14, 3.23) | 0.393 (3.14, 3.24) | < 0.001 (3.1, 3.49) | < 0.001 (3.11, 3.46) |
| *ADI* | < 0.001 (38.46, 45.52) | < 0.001 (38.39, 45.81) | < 0.001 (38.41, 43.82) | < 0.001 (37.66, 47.39) | < 0.001 (37.88, 46.12) |
| *Education* | < 0.001 (15.39, 14.14) | < 0.001 (15.39, 14.18) | < 0.001 (15.39, 14.46) | < 0.001 (15.43, 14.63) | < 0.001 (15.44, 14.44) |
| *INR* | < 0.001 (3.75, 2.67) | < 0.001 (3.76, 2.67) | < 0.001 (3.75, 3.03) | < 0.001 (3.8, 3) | < 0.001 (3.79, 3.01) |
| *PRFQ* | < 0.001 (0.42, 0.72) | < 0.001 (0.42, 0.73) | < 0.001 (0.42, 0.7) | < 0.001 (0.41, 0.67) | < 0.001 (0.41, 0.65) |

Table S1

This table provides P values from t tests comparing the baseline SES and 6-month mental health data between participants and non-participants at each time point after 6 months. Mean values for the non-dropouts and the dropouts are provided in parentheses in that order. The table shows that SES and attention symptoms significantly differed between non-dropouts and dropouts. INR = income-to-needs ratio, EDU = educational attainment, PRFQ = parent-reported financial adversity, ADI = area deprivation index

## **Table S2: Associations between SES and trajectories of mental health across the whole sample (non-sex-stratified results)**

| **Mental health variable** | **SES Variable** | **B** | **SE** | **T** | **p** |  |
| --- | --- | --- | --- | --- | --- | --- |
| Internalizing | Income-to-needs | 0.024 | 0.005 | 4.546 | < 0.001 | * |
|  | Education | 0.007 | 0.005 | 1.419 | 0.156 |  |
|  | Low material hardship | 0.0003 | 0.004 | 0.081 | 0.935 |  |
|  | Neighborhood advantage | 0.010 | 0.005 | 2.023 | 0.043 |  |
| Externalizing | Income-to-needs | 0.016 | 0.005 | 3.167 | 0.002 | * |
|  | Education | -0.006 | 0.005 | -1.170 | 0.242 |  |
|  | Low material hardship | 0.002 | 0.004 | 0.389 | 0.697 |  |
|  | Neighborhood advantage | 0.002 | 0.005 | 0.489 | 0.625 |  |
| Attention | Income-to-needs | 0.018 | 0.005 | 3.779 | < 0.001 | * |
|  | Education | 0.004 | 0.005 | 0.747 | 0.455 |  |
|  | Low material hardship | -0.001 | 0.004 | -0.306 | 0.760 |  |
|  | Neighborhood advantage | 0.006 | 0.005 | 1.250 | 0.211 |  |

Table S2. Model output for independent associations between SES indicators and internalizing, externalizing, and attention symptom trajectories. Results are from models where all four SES indicators were included simultaneously. The table reports uncorrected p values. * p < 0.0167

ADI = area deprivation index, EDU = educational attainment in years, INR = income-to-needs ratio, MD = material hardship

## **Figure S6: Sex differences in mental health trajectories (Figure S6)**


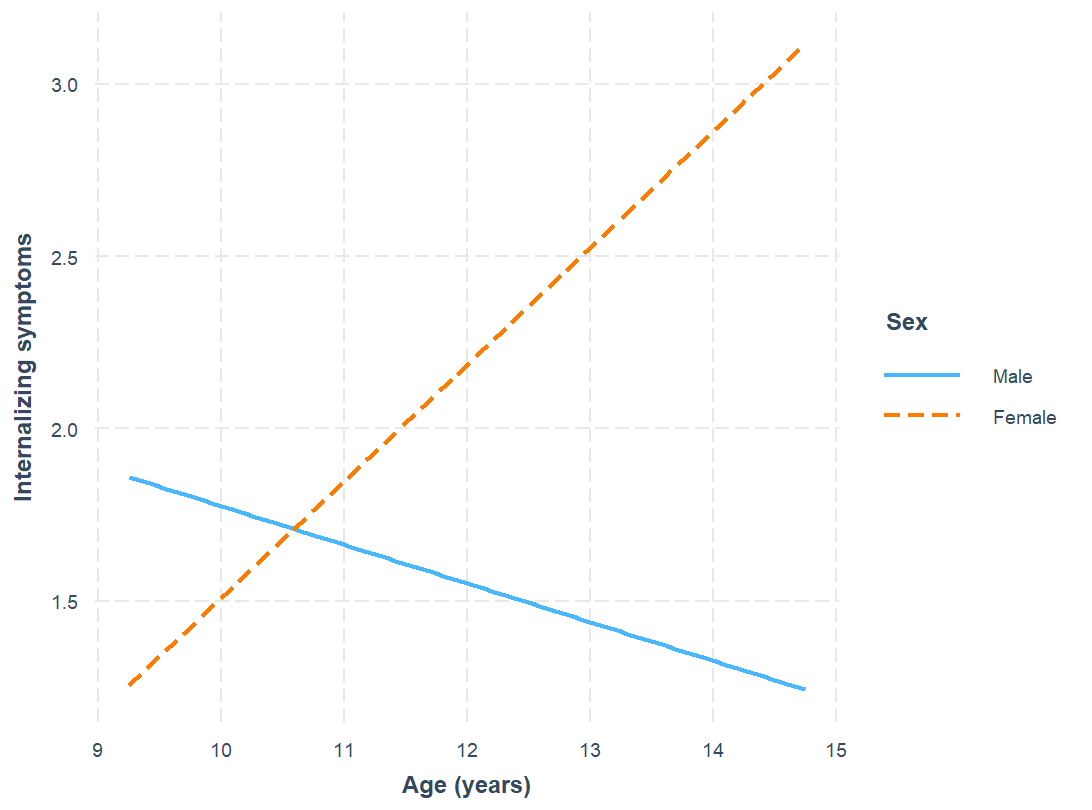

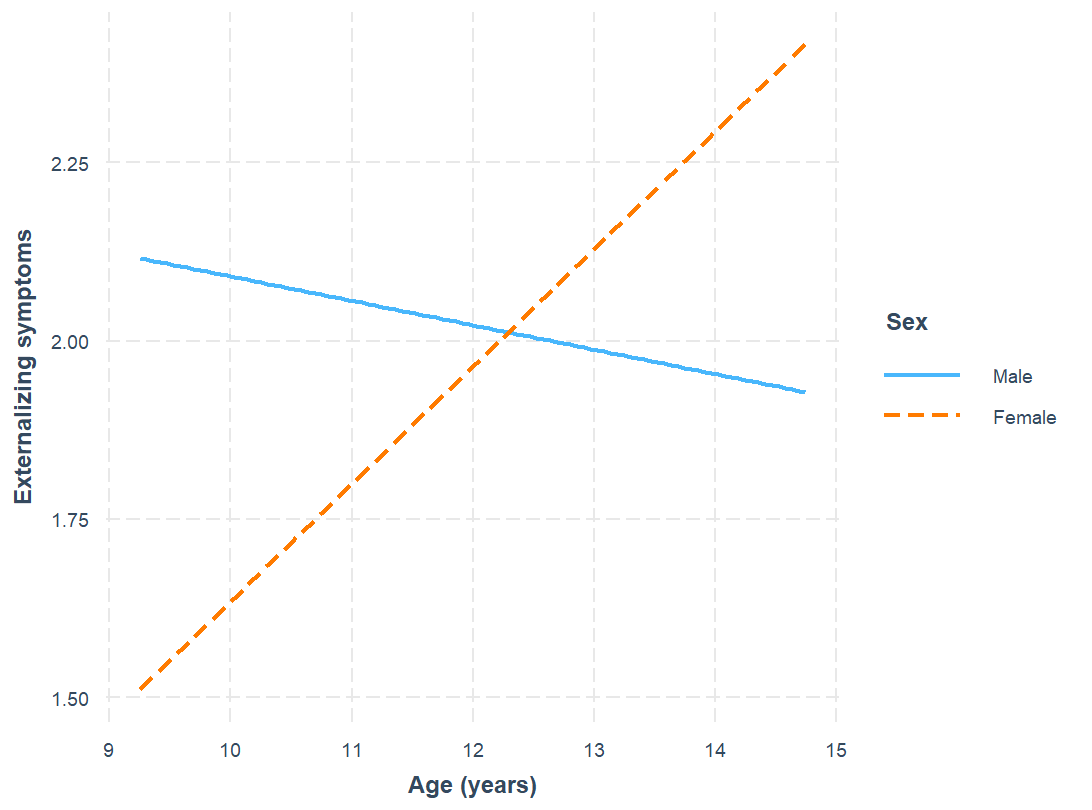

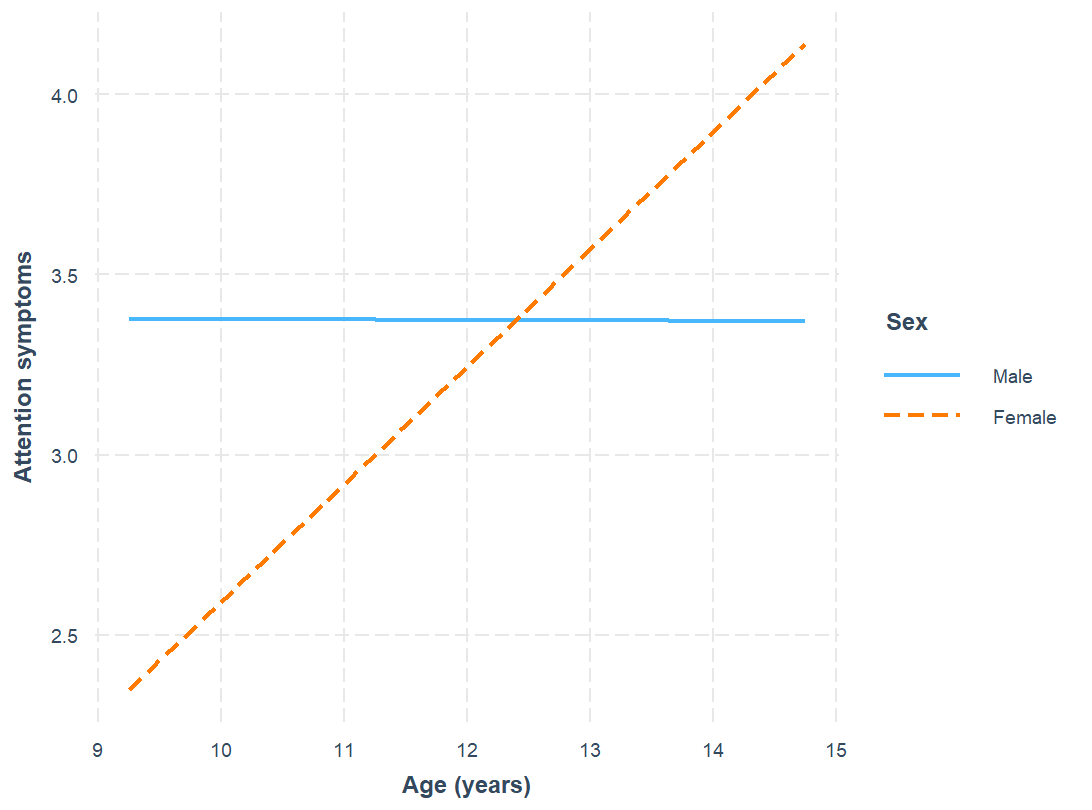


Figure S6

## **Simple slope results for main models**

***SIMPLE SLOPES ANALYSIS FOR INTERNALIZING MODEL IN FEMALES***

Slope of age when inr = -0.95498173 (- 1 SD):

Est. S.E. t val. p

------ ------ -------- ------

0.11 0.01 10.07 0.00

Slope of age when inr = 0.04000785 (Mean):

Est. S.E. t val. p

------ ------ -------- ------

0.15 0.01 19.59 0.00

Slope of age when inr = 1.03499744 (+ 1 SD):

Est. S.E. t val. p

------ ------ -------- ------

0.18 0.01 18.12 0.00

***SIMPLE SLOPES ANALYSIS FOR EXTERNALIZING MODEL IN MALES***

Slope of age when inr = -0.93887974 (- 1 SD):

Est. S.E. t val. p

------- ------ -------- ------

-0.03 0.01 -2.89 0.00

Slope of age when inr = 0.04858715 (Mean):

Est. S.E. t val. p

------- ------ -------- ------

-0.01 0.01 -1.14 0.25

Slope of age when inr = 1.03605404 (+ 1 SD):

Est. S.E. t val. p

------ ------ -------- ------

0.01 0.01 1.46 0.14

***SIMPLE SLOPES ANALYSIS FOR ATTENTION MODEL IN MALES***

Slope of age when inr = -0.93703745 (- 1 SD):

Est. S.E. t val. p

------- ------ -------- ------

-0.02 0.01 -2.46 0.01

Slope of age when inr = 0.05004864 (Mean):

Est. S.E. t val. p

------- ------ -------- ------

-0.00 0.01 -0.20 0.84

Slope of age when inr = 1.03713473 (+ 1 SD):

Est. S.E. t val. p

------ ------ -------- ------

0.02 0.01 2.38 0.02

## **Table S3: Model output excluding race-associated change as a covariate**

mental_health (time varying) ~ age + adi*age + inr*age + edu*age + prfq*age + adi + inr + edu + prfq + (1|family) + (1|subject)

**Males**

| *SES Variable* | *Mental health variable* | *B* | *SE* | *T* | *P* |
| --- | --- | --- | --- | --- | --- |
| *ADI* | *Internalizing* | 0.010 | 0.006 | 1.550 | 0.121 |
| *ADI* | *Externalizing* | 0.010 | 0.006 | 1.662 | 0.097 |
| *ADI* | *Attention* | 0.011 | 0.006 | 1.839 | 0.066 |
| *EDU* | *Internalizing* | 0.012 | 0.007 | 1.728 | 0.084 |
| *EDU* | *Externalizing* | -0.011 | 0.007 | -1.682 | 0.093 |
| *EDU* | *Attention* | 0.004 | 0.006 | 0.627 | 0.531 |
| *INR* | *Internalizing* | 0.017 | 0.007 | 2.434 | 0.015 |
| *INR* | *Externalizing* | 0.022 | 0.007 | 3.206 | 0.001* |
| *INR* | *Attention* | 0.025 | 0.007 | 3.745 | 0.000* |
| *PRFQ* | *Internalizing* | 0.002 | 0.006 | 0.275 | 0.783 |
| *PRFQ* | *Externalizing* | 0.001 | 0.006 | 0.184 | 0.854 |
| *PRFQ* | *Attention* | 0.001 | 0.005 | 0.157 | 0.875 |

**Females**

| *SES Variable* | *Mental health variable* | *B* | *SE* | *T* | *P* |
| --- | --- | --- | --- | --- | --- |
| *ADI* | *Internalizing* | 0.028 | 0.007 | 4.218 | < 0.001* |
| *ADI* | *Externalizing* | 0.012 | 0.006 | 1.837 | 0.066 |
| *ADI* | *Attention* | 0.016 | 0.006 | 2.496 | 0.013* |
| *EDU* | *Internalizing* | 0.001 | 0.007 | 0.182 | 0.856 |
| *EDU* | *Externalizing* | -0.013 | 0.007 | -1.878 | 0.060 |
| *EDU* | *Attention* | -0.002 | 0.007 | -0.321 | 0.748 |
| *INR* | *Internalizing* | 0.037 | 0.008 | 4.888 | < 0.001* |
| *INR* | *Externalizing* | 0.005 | 0.007 | 0.664 | 0.507 |
| *INR* | *Attention* | 0.014 | 0.007 | 2.015 | 0.044 |
| *PRFQ* | *Internalizing* | 0.005 | 0.006 | 0.823 | 0.410 |
| *PRFQ* | *Externalizing* | 0.007 | 0.006 | 1.238 | 0.216 |
| *PRFQ* | *Attention* | 0.002 | 0.006 | 0.320 | 0.749 |

Table S3. The table reports uncorrected p values.

* *p* < 0.0167

ADI = area deprivation index, EDU = educational attainment in years, INR = income-to-needs ratio, PRFQ = parent reported financial adversity

## **Table S4: Model output for all 3-way interactions excluding race-associated change as a covariate**

mental_health (time varying) ~ age + SES1*SES2*age + SES1*age + SES2*age + SES1 + SES2 + (1|family) + (1|subject)

**Males**

| Mental health variable | 3-way interaction | B | SE | T | p |
| --- | --- | --- | --- | --- | --- |
| Internalizing | INR*EDU | -0.012 | 0.006 | -2.062 | 0.039 |
| Internalizing | EDU*PRFQ | -0.006 | 0.007 | -0.797 | 0.425 |
| Internalizing | ADI*PRFQ | -0.005 | 0.005 | -0.997 | 0.319 |
| Internalizing | ADI*INR | -0.005 | 0.006 | -0.769 | 0.442 |
| Internalizing | ADI*EDU | -0.007 | 0.006 | -1.201 | 0.230 |
| Externalizing | INR*EDU^1^ | -0.010 | 0.006 | -1.771 | 0.076 |
| Externalizing | EDU*PRFQ | -0.015 | 0.007 | -2.185 | 0.029 |
| Externalizing | ADI*PRFQ | -0.007 | 0.005 | -1.250 | 0.211 |
| Externalizing | ADI*INR | -0.009 | 0.006 | -1.466 | 0.143 |
| Externalizing | ADI*EDU | -0.008 | 0.006 | -1.436 | 0.151 |
| Attention | INR*EDU | -0.012 | 0.005 | -2.248 | 0.025 |
| Attention | EDU*PRFQ | -0.012 | 0.006 | -1.899 | 0.058 |
| Attention | ADI*PRFQ | -0.006 | 0.005 | -1.182 | 0.237 |
| Attention | ADI*INR | -0.014 | 0.006 | -2.518 | 0.012 |
| Attention | ADI*EDU | -0.006 | 0.006 | -1.076 | 0.282 |

**Females**

| Mental health variable | 3-way interaction | B | SE | T | p |
| --- | --- | --- | --- | --- | --- |
| Internalizing | INR*EDU | -0.005 | 0.006 | -0.900 | 0.368 |
| Internalizing | EDU*PRFQ | 0.013 | 0.007 | 1.790 | 0.074 |
| Internalizing | ADI*PRFQ | -0.004 | 0.006 | -0.667 | 0.505 |
| Internalizing | ADI*INR | -0.014 | 0.006 | -2.281 | 0.023 |
| Internalizing | ADI*EDU | -0.019 | 0.006 | -3.012 | 0.003 |
| Externalizing | INR*EDU^1^ | 0.002 | 0.006 | 0.298 | 0.766 |
| Externalizing | EDU*PRFQ | -0.003 | 0.007 | -0.357 | 0.721 |
| Externalizing | ADI*PRFQ | -0.018 | 0.005 | -3.326 | 0.001 |
| Externalizing | ADI*INR | -0.012 | 0.006 | -2.045 | 0.041 |
| Externalizing | ADI*EDU | -0.009 | 0.006 | -1.455 | 0.146 |
| Attention | INR*EDU | -0.007 | 0.006 | -1.266 | 0.206 |
| Attention | EDU*PRFQ | 0.001 | 0.007 | 0.100 | 0.921 |
| Attention | ADI*PRFQ | -0.007 | 0.005 | -1.407 | 0.160 |
| Attention | ADI*INR | -0.004 | 0.006 | -0.652 | 0.514 |
| Attention | ADI*EDU | -0.003 | 0.006 | -0.549 | 0.583 |

Table S4. The table reports uncorrected p values. ADI = area deprivation index, EDU = educational attainment in years, INR = income-to-needs ratio, PRFQ = parent reported financial adversity

## **Table S5: Model output for independent effects (with only one SES indicator in the model)**

Model equation: mental_health (time varying) ~ age + SES_var*age + race_ethnicity*age + race_ethnicity + SES_var + (1|family) + (1|subject)

**Males**

| *SES Variable* | *Mental health variable* | *B* | *SE* | *T* | *P* |
| --- | --- | --- | --- | --- | --- |
| *INR* | *Internalizing* | 0.021 | 0.006 | 3.678 | < 0.001* |
| *INR* | *Externalizing* | 0.017 | 0.006 | 3.123 | 0.002* |
| *INR* | *Attention* | 0.026 | 0.005 | 4.897 | < 0.001* |
| *EDU* | *Internalizing* | 0.020 | 0.006 | 3.324 | 0.001* |
| *EDU* | *Externalizing* | 0.003 | 0.006 | 0.493 | 0.622 |
| *EDU* | *Attention* | 0.018 | 0.006 | 3.229 | 0.001* |
| *PRFQ* | *Internalizing* | 0.007 | 0.006 | 1.168 | 0.243 |
| *PRFQ* | *Externalizing* | 0.002 | 0.005 | 0.446 | 0.656 |
| *PRFQ* | *Attention* | 0.007 | 0.005 | 1.300 | 0.193 |
| *ADI* | *Internalizing* | 0.014 | 0.006 | 2.346 | 0.019 |
| *ADI* | *Externalizing* | 0.008 | 0.006 | 1.351 | 0.177 |
| *ADI* | *Attention* | 0.014 | 0.006 | 2.602 | 0.009* |

**Females**

| *SES Variable* | *Mental health variable* | *B* | *SE* | *T* | *P* |
| --- | --- | --- | --- | --- | --- |
| *INR* | *Internalizing* | 0.044 | 0.006 | 7.266 | < 0.001* |
| *INR* | *Externalizing* | 0.013 | 0.006 | 2.213 | 0.027 |
| *INR* | *Attention* | 0.019 | 0.006 | 3.304 | < 0.001* |
| *EDU* | *Internalizing* | 0.028 | 0.006 | 4.412 | < 0.001* |
| *EDU* | *Externalizing* | 0.006 | 0.006 | 1.031 | 0.302 |
| *EDU* | *Attention* | 0.012 | 0.006 | 2.084 | 0.037 |
| *PRFQ* | *Internalizing* | 0.017 | 0.006 | 2.794 | 0.005* |
| *PRFQ* | *Externalizing* | 0.010 | 0.006 | 1.739 | 0.082 |
| *PRFQ* | *Attention* | 0.006 | 0.006 | 1.126 | 0.260 |
| *ADI* | *Internalizing* | 0.032 | 0.006 | 5.166 | < 0.001* |
| *ADI* | *Externalizing* | 0.007 | 0.006 | 1.255 | 0.209 |
| *ADI* | *Attention* | 0.015 | 0.006 | 2.528 | 0.011* |

Table S5. The table reports uncorrected p values.

** p*FDR < 0.0167

ADI = area deprivation index, EDU = educational attainment in years, INR = income-to-needs ratio, PRFQ = parent reported financial adversity

## **Table S6: Results of associations between income-to-needs and change in mental health (with no covariates)**

Higher INR was associated with greater increases in mental health problems across all domains

mental_health (time varying) ~ INR* age + (1|family) + (1|subject)

| *SES Variable* | *Mental health variable* | *B* | *SE* | *T* | *P* |
| --- | --- | --- | --- | --- | --- |
| *INR* | *Internalizing (females)* | 0.053 | 0.006 | 9.615 | < 0.001 |
| *INR* | *Externalizing (males)* | 0.022 | 0.005 | 4.266 | < 0.001 |
| *INR* | *Attention (males)* | 0.033 | 0.005 | 6.769 | < 0.001 |

Table S6. The table reports uncorrected p values. INR = income-to-needs ratio

## **Table S7: Associations between INR and trajectories of mental health using untransformed (i.e., raw) data**

Higher INR was associated with greater increases in internalizing and attention symptoms

| *SES Variable* | *Mental health variable* | *B* | *SE* | *T* | *P* |
| --- | --- | --- | --- | --- | --- |
| *INR* | *Internalizing (females)* | 0.063 | 0.017 | 3.652 | < 0.001 |
| *INR* | *Externalizing (males)* | 0.034 | 0.014 | 2.530 | 0.011 |
| *INR* | *Attention (males)* | 0.068 | 0.017 | 3.905 | < 0.001 |

Table S7. ADI = area deprivation index, EDU = educational attainment in years, INR = income-to-needs ratio, PRFQ = parent reported financial adversity

* *p* < 0.0167

## **Results of associations between income-to-needs and COVID-19 impact**

To help interpret findings, we examined associations between INR and self-reported COVID-19 life impact and changes in familial and social relationships. These additional data were gathered through a dedicated COVID-19 sub-study conducted during the pandemic period. We used data from the first wave of data collection. We included age, sex, and race/ethnicity as covariates.

We found that INR was positively associated with self-reported life impact (“How much do you think your life has changed due to coronavirus”). Adolescents from higher INR households reported greater changes in their life due to the pandemic (B = 0.086, SE = 0.018, p < .001). Higher INR was associated with worsening communication with parents (B = -0.046, SE = 0.012, p < .001) but not friends (B = 0.009, SE = 0.013, p = .467). Higher INR was also associated with more negative changes in familial relationship quality (B = -0.035, SE = 0.012, p = .001), but not relationship quality with friends. INR was also negatively associated with the number of days parents were involved with adolescents’ schoolwork (B = -0.014, SE = 0.067, p = .034).

## **Table S8: Model output for all 3-way interactions**

No associations survived correction for multiple comparisons (*pFDR* < 0.0167).

**Males**

| Mental health variable | Interaction | B | SE | T | P value |
| --- | --- | --- | --- | --- | --- |
| Internalizing | INR*EDU^1^ | -0.008 | 0.006 | -1.354 | 0.176 |
| Internalizing | EDU*PRFQ | -0.006 | 0.007 | -0.895 | 0.371 |
| Internalizing | ADI*PRFQ | -0.005 | 0.005 | -0.843 | 0.399 |
| Internalizing | ADI*INR | 0.001 | 0.006 | 0.241 | 0.810 |
| Internalizing | ADI*EDU | -0.003 | 0.006 | -0.495 | 0.620 |
| Externalizing | INR*EDU^1^ | -0.009 | 0.006 | -1.646 | 0.100 |
| Externalizing | EDU*PRFQ | -0.017 | 0.007 | -2.458 | 0.014 |
| Externalizing | ADI*PRFQ | -0.005 | 0.005 | -0.955 | 0.340 |
| Externalizing | ADI*INR | -0.003 | 0.006 | -0.555 | 0.579 |
| Externalizing | ADI*EDU | -0.005 | 0.006 | -0.856 | 0.392 |
| Attention | INR*EDU | -0.009 | 0.005 | -1.732 | 0.083 |
| Attention | EDU*PRFQ | -0.013 | 0.006 | -2.072 | 0.038 |
| Attention | ADI*PRFQ | -0.005 | 0.005 | -0.918 | 0.359 |
| Attention | ADI*INR | -0.008 | 0.006 | -1.474 | 0.141 |
| Attention | ADI*EDU | -0.002 | 0.006 | -0.315 | 0.753 |

**Females**

| Mental health variable | Interaction | B | SE | T | P value |
| --- | --- | --- | --- | --- | --- |
| Internalizing | INR*EDU | -0.003 | 0.006 | -0.488 | 0.626 |
| Internalizing | EDU*PRFQ | 0.010 | 0.007 | 1.378 | 0.168 |
| Internalizing | ADI*PRFQ | -0.002 | 0.006 | -0.364 | 0.716 |
| Internalizing | ADI*INR | -0.007 | 0.006 | -1.061 | 0.289 |
| Internalizing | ADI*EDU | -0.013 | 0.006 | -2.142 | 0.032 |
| Externalizing | INR*EDU^1^ | -0.003 | 0.006 | -0.600 | 0.549 |
| Externalizing | EDU*PRFQ | -0.004 | 0.007 | -0.574 | 0.566 |
| Externalizing | ADI*PRFQ | -0.014 | 0.005 | -2.628 | 0.009 |
| Externalizing | ADI*INR | -0.010 | 0.006 | -1.670 | 0.095 |
| Externalizing | ADI*EDU | -0.006 | 0.006 | -1.075 | 0.283 |
| Attention | INR*EDU | -0.007 | 0.006 | -1.156 | 0.248 |
| Attention | EDU*PRFQ | -0.001 | 0.007 | -0.101 | 0.920 |
| Attention | ADI*PRFQ | -0.005 | 0.005 | -0.986 | 0.324 |
| Attention | ADI*INR | 0.002 | 0.006 | 0.295 | 0.768 |
| Attention | ADI*EDU | 0.001 | 0.006 | 0.174 | 0.862 |

Table S8. Each interaction term also included an interaction with age to examine changes over time. The table reports uncorrected p values.

ADI = area deprivation index, EDU = educational attainment in years, INR = income-to-needs ratio, PRFQ = parent reported financial adversity

## **Table S9: Model output for the association between baseline SES and 6-month follow-up mental health symptoms**

**Males**

| *SES Variable* | *Mental health variable* | *B* | *SE* | *T* | *P* |
| --- | --- | --- | --- | --- | --- |
| *INR* | *Internalizing* | -0.025 | 0.020 | -1.290 | 0.197 |
| *EDU* | *Internalizing* | -0.050 | 0.019 | -2.589 | 0.010 |
| *PRFQ* | *Internalizing* | -0.073 | 0.016 | -4.590 | < 0.001 |
| *ADI* | *Internalizing* | -0.040 | 0.018 | -2.206 | 0.027 |
| *INR* | *Externalizing* | -0.063 | 0.020 | -3.173 | 0.002 |
| *EDU* | *Externalizing* | -0.012 | 0.020 | -0.611 | 0.541 |
| *PRFQ* | *Externalizing* | -0.097 | 0.016 | -6.120 | < 0.001 |
| *ADI* | *Externalizing* | -0.044 | 0.018 | -2.399 | 0.016 |
| *INR* | *Attention* | -0.018 | 0.019 | -0.957 | 0.338 |
| *EDU* | *Attention* | -0.062 | 0.018 | -3.384 | 0.001 |
| *PRFQ* | *Attention* | -0.092 | 0.015 | -6.113 | < 0.001 |
| *ADI* | *Attention* | -0.031 | 0.017 | -1.765 | 0.078 |

**Females**

| *SES Variable* | *Mental health variable* | *B* | *SE* | *T* | *P* |
| --- | --- | --- | --- | --- | --- |
| *INR* | *Internalizing* | -0.105 | 0.021 | -5.103 | < 0.001 |
| *EDU* | *Internalizing* | -0.020 | 0.020 | -0.971 | 0.331 |
| *PRFQ* | *Internalizing* | -0.068 | 0.017 | -4.036 | < 0.001 |
| *ADI* | *Internalizing* | -0.033 | 0.019 | -1.751 | 0.080 |
| *INR* | *Externalizing* | -0.105 | 0.021 | -5.042 | < 0.001 |
| *EDU* | *Externalizing* | 0.027 | 0.020 | 1.315 | 0.189 |
| *PRFQ* | *Externalizing* | -0.058 | 0.017 | -3.422 | 0.001 |
| *ADI* | *Externalizing* | -0.050 | 0.019 | -2.597 | 0.009 |
| *INR* | *Attention* | -0.052 | 0.020 | -2.580 | 0.010 |
| *EDU* | *Attention* | -0.067 | 0.020 | -3.359 | 0.001 |
| *PRFQ* | *Attention* | -0.062 | 0.017 | -3.743 | < 0.001 |
| *ADI* | *Attention* | -0.045 | 0.019 | -2.406 | 0.016 |

Table S9. The table reports uncorrected p values. ADI = area deprivation index, EDU = educational attainment in years, INR = income-to-needs ratio, PRFQ = parent reported financial adversity. Model accounts for race/ethnicity.

## References

1. Barch DM, Albaugh MD, Baskin-Sommers A, et al. Demographic and Mental Health Assessments in the Adolescent Brain and Cognitive Development Study: Updates and Age-Related Trajectories. *Developmental Cognitive Neuroscience*. Published online October 29, 2021:101031. doi:10.1016/J.DCN.2021.101031
